# Antimicrobial susceptibility pattern of aerobic bacteria responsible for post-surgical wound infection of the patients admitted into Khulna Medical College Hospital, Bangladesh

**DOI:** 10.1099/acmi.0.000401

**Published:** 2024-02-13

**Authors:** Arrafy Rahman, Khondoker Moazzem Hossain, Shithima Sayed, S. M. Tushar Alam, Omar Faruq, Md. Ahasun Habib, Fahmida Khatun

**Affiliations:** ^1^​ University of Auckland, Auckland, New Zealand; ^2^​ Department of Ophthalmology, College of Medicine, Chung-Ang University Hospital, Seoul, South Korea; ^3^​ Biotechnology and Genetic Engineering Discipline, Life Science School, Khulna University, Khulna-9208, Bangladesh; ^4^​ Khulna Medical College Hospital, Khulna, Bangladesh; ^5^​ Biomedical Engineering Doctoral Program, Boise State University, Boise, ID, USA; ^6^​ Department of Biotechnology, Bangladesh Agricultural University, Mymensingh-2202, Bangladesh

**Keywords:** antimicrobial agent, infection, resistance, surgical wound, susceptibility

## Abstract

Resistance against antimicrobial agents is dramatically increasing and gradually impacting treatment costs. Using existing drugs would have helped avoid bacterial infections in various circumstances. The primary objectives of this study were to determine the prevalence of pathogens responsible for postsurgical wound infections and their antimicrobial susceptibility and resistance pattern among the patients admitted to Khulna Medical College Hospital, Khulna Bangladesh. This cross-sectional study involved 250 patients suffering from postsurgical wound infection as respondents. The bacterial pathogens were isolated from pus samples obtained from those patients. The isolated bacterial pathogens were identified through several standard biochemical tests, and finally, the culture sensitivity tests of those bacterial isolates were performed. The study was conducted from August 2019 to June 2020. Data regarding the patient’s age, gender, occupation, surgery performed, duration of hospital stay, and comorbidity were also documented using standard questionnaires. Five bacterial pathogens were identified with different frequencies, including *Pseudomonas aeruginosa* (36 %), *Escherichia coli* (21.2 %), *Staphylococcus aureus* (8.8 %), *Klebsiella* spp. (7.2 %) and *Proteus* spp. (4.8 %). These bacterial pathogens showed sensitivity to ciprofloxacin (75 %), piperacillin-tazobactam (56.7 %) and gentamicin (50 %). Besides, *S. aureus* showed sensitivity to linezolid and vancomycin and resistance to cefuroxime, ceftazidime and imipenem. Male patients (68.4 %) suffered more from postsurgical wound infection than female patients (31.6 %). Patients aged 31 to 40 years were more severely affected than patients from other age groups. Postsurgical wound infection was vigorously observed in the patients who underwent hand surgery. Intensive occurrence of this infection was found in the patients who stayed in the hospital from 31 to 40 days. Diabetic patients suffered more from postsurgical wound infection compared to the other patients. Throughout the study, ciprofloxacin has been the best performer against *E. coli*, *Klebsiella* spp., and *Proteus* spp., and gentamicin showed better performance against *S. aureus*. The antibiotic resistance pattern of these bacterial pathogens reflects the worldwide necessity of rational antibiotic management and proper steps to maintain hospital hygiene in Bangladesh.

## Introduction

Post-surgical wound infection or secondary wound infection may be defined as an infection that occurs about 30 days after surgery. It may be related to the surgery itself or the postsurgical course. This type of infection is one of the major complications in third-world countries, leading to an increase in mortality and morbidity. Such infections start with the attachment of micro-organisms to the host tissue and subsequently lead to pus formation. These infections not only cause diverse complications after surgery but also increase medical expenses and the risk of death [1]. Most of the postsurgical or secondary wound infections are originated from unhygienic hospital environments, which vary from one to another. One of the most challenging factors is the need for ideal criteria for diagnosing and monitoring these infections’ epidemiology. Antimicrobial therapy may be a better choice to prevent the growth of micro-organisms in the wound sites. Although new antibiotics are available, there is still a threat of antibiotic resistance in bacterial pathogens due to the extensive use of prophylactic antibiotics [1].

A wide range of micro-organisms infects the surgical sites in a number of manners. In postsurgical infection, the prevalence of *Staphylococcus aureus* is most severe (30–40 %) and *Pseudomonas aeruginosa* is 5–15 %. *Enterococci* and *Enterobacteriaceae* have also been found in postsurgical infections. In Ethiopia, *S. aureus*, *Klebsiella* species, *Escherichia coli*, *Proteus* species, *Streptococcus* species, *Enterobacter* species, *Pseudomonas* species and coagulase negative *Staphylococci* were reported as the most common pathogens found in postsurgical infections [2]. The study conducted in Bangladesh showed that most of the bacteria isolated from postsurgical infections were *S. aureus, E. coli*, *Klebsiella pneumoniae*, *Acinetobacter* species, *P. aeruginosa*, *Proteus* species and *Citrobacter* species [3]. Antimicrobial agents are good candidates for curative measures against microbial infections. The use of antimicrobial agents over time to treat postsurgical infections causes selective pressure on bacterial populations. In this way, the repetitive use of antibiotics ultimately leads to the emergence and spread of antimicrobial-resistant bacterial pathogens. Antibacterial resistance has increased in developed and developing countries, which is a significant threat to public health [4]. This worldwide public health problem is getting more and more dangerous in developing countries, where the infection load is high, and the use of antimicrobials is excessive [5].

In most developing countries like Bangladesh, antibiotic drugs are readily available to patients. They can obtain any antibiotic from the pharmaceutical counters with or without a prescription from the medical practitioners. However, poor or improper prescribing practices leading to irrational and unnecessary use of antimicrobials and the proliferation of counterfeit drugs are also responsible for the emergence of resistance among bacterial organisms [6]. In addition, the gradual increase in antibacterial resistance has made therapeutic measures more complex, lengthy and expensive [7].

Several studies on the micro-organisms causing postsurgical infections and the pattern of their antibiotic susceptibility have been carried out in different countries of Asia and even in various Government Medical College Hospitals in Bangladesh. However, no such study has been conducted in Khulna Medical College Hospital, Khulna. Therefore, in the current study, we tried to investigate the bacterial frequency in surgical wound infections in Khulna Medical College Hospital, Khulna, and find out the antibiotic susceptibility pattern of those bacterial pathogens involved in the surgical wound infections.

## Methods

### Study area

The study was conducted at the Khulna Medical College Hospital, Khulna, Bangladesh and the Science View Diagnostic and Research Center, Sonadanga, Khulna. The pus samples were collected from Khulna Medical College Hospital and tested in the Science View Diagnostic and Research Center. The study was carried out over the period from August 2019 to June 2020.

### Study population

In this work, the patients admitted to Khulna Medical College Hospital, Khulna, were considered as the study population. The pus samples were collected from the surgical sites of 250 patients suffering from wound infections at surgical sites for about 30 days of postsurgery. The presence of cellulitis, contaminated wounds, suture abscesses and patients who were taking antibiotics were excluded from this study. The study population included 171 males (68.4 %) and 79 (31.6 %) females with different age ranges. Moreover, some patients had diabetes, asthma, arthritis, stroke, heart attack, ulcer and jaundice alongside postsurgical infections. The patient information regarding age, gender, surgery and types of wounds were obtained from their case records.

### Collection of samples

Pus samples were collected from the surgical sites of the patients with wound infections using the standard collection technique. In brief, a sterile cotton wool swab stick was used to take the pus sample from the wound site. The swab sticks were applied smoothly into the surgical wound sites and rotated around the area to properly collect the pus sample. Immediately after collecting, the pus sample was transferred to the sterile laboratory test tubes. The test tubes were labelled correctly and carried to the laboratory for further investigations.

### Identification of bacterial isolates

The collected wound swabs (precisely the pus samples) were first inoculated onto freshly prepared blood agar medium and McConkey agar medium. Next, the media containing the pus samples were incubated at 37 °C overnight for bacterial growth. After overnight incubation, the type and colour of the bacterial colonies were closely observed, and the colony characteristics were recorded accurately. Finally, the bacterial isolates were identified through standard appropriate morphological and biochemical tests, including Gram staining, Oxidase test, Coagulase test and Catalase test.

Briefly, in the Gram-staining test, distilled water was dropped on a slide and smeared by a colony from the 24 h culture. After heat fixation, a drop of crystal violet was added for 60 s and rinsed immediately. Next, iodine was added for 60 s, rinsing, dipping 70 % ethanol for 10–20 s, and rinsing quickly to avoid decolorization. Eventually, the added secondary stain was allowed for 2 min, rinsed and dried for microscopic observation.

### Antibiotic susceptibility tests

The susceptibility of antibiotic testing was done on the Muller–Hinton agar medium by disc diffusion. *E. coli* (ATCC 25922) *P. aeruginosa* (ATCC 27853), *S. aureus* (ATCC 29213), *K. pneumonia* (ATCC 700603) and *P. vulgaris* (ATCC29905) were used as the control strains to ensure the reproducibility and accuracy of the antibiotic susceptibility test. The selection of antimicrobial agents and their inhibition zones were measured according to CLSI guidelines (Table 1) [8].

**Table 1.** Antibiotics used in the disc-diffusion method

**Antibiotics used against *E. coli***	Amoxicillin, cephradine, co-trimoxazole, ciprofloxacin, nalidixic acid, ceftriaxone, gentamicin, amoxicillin+clavulanic acid, ceftazidime, cefuroxime, amikacin, imipenem, netilmicin, piperacillin–tazobactam (TZP) and meropenem
**Antibiotics used against *Pseudomonas***	Ciprofloxacin, gentamicin, ceftazidime, amikacin, imipenem, netilmicin, piperacillin- tazobactam (TZP), colistin, cefepime and meropenem
**Antibiotics used against *S. aureus***	Amoxicillin, cephradine, co-trimoxazole, ciprofloxacin, ceftriaxone, gentamicin, amoxicillin+clavulanic acid, ceftazidime, cefuroxime, amikacin, imipenem, netilmicin, piperacillin-tazobactam (TZP), meropenem, linezolid, levofloxacin and vancomycin
**Antibiotics used against *Klebsiella* spp**.	Amoxicillin, cephradine, co-trimoxazole, ciprofloxacin, ceftriaxone, gentamicin, amoxicillin+clavulanic acid, ceftazidime, cefuroxime, amikacin, imipenem, netilmicin, cefotaxime and nalidixic acid
**Antibiotics used against *Proteus* spp.**	Amoxicillin, cephradine, co-trimoxazole, ciprofloxacin, ceftriaxone, gentamicin, amoxicillin+clavulanic acid, ceftazidime, cefuroxime, amikacin, imipenem, netilmicin, cefotaxime, nalidixic acid, colistin, meropenem and cefepime

### Data management and analysis

The collected data were entered into an Excel spreadsheet in a coded form and analysed using SPSS version 21 on a password-protected computer. Data were summarized using descriptive statistics.

## Results

The patients of Khulna Medical College Hospital suffering from postsurgical wound infection were considered this study’s respondents. The data regarding gender, age, occupation, surgery performed, duration of hospital stay and comorbidity were collected. In this study, 68.4 % of the respondents were male, and 31.6 % were female. A total of 14 % of respondents had undergone abdominal surgery, including liver surgery, stomach surgery, pancreatic surgery, intestinal surgery, colonic surgery and gall bladder surgery. Leg surgery was performed in the case of 16.8 % of respondents, and 17.6 % of the patients accounted for hand surgery. Besides, 8.4 % of the respondents had herniorrhaphy, and 14 % of respondents had a craniotomy. Vascular surgery involved 10.4 % of the respondents and the joint prosthesis was carried out in the case of 10.8 % of the respondents. A total of 8 % of the respondents accounted for skin surgery. Among the respondents with comorbidities, 4 % suffered from diabetes, 2.8 % suffered from asthma, 1.2 % had arthritic complications, 1.2 % went through stroke, 1.6 % had heart blocks, 0.8 % suffered from ulcers and 1.2 % suffered from jaundice. The patients aged 31–40 years showed the highest frequency of postsurgical wound infection (19.2 %). Postsurgical wound infection was vigorously observed in the patients who stayed in the hospital from 31 to 40 days postsurgery (36.4 %).

Our current study showed the total number of positive bacterial growth and no growth among 250 respondents, where 195 showed positive bacterial growth and 55 showed no change.

This study has isolated five different species of bacterial pathogens (Table 2). They are *E. coli*, *P. aeruginosa*, *S. aureus*, *Klebsiella* spp. and *Proteus* spp. The bacteria colony in various media showed distinct colour characteristics, which helped to identify the specific infection. In McConkey agar medium, *P. aeruginosa*, *E. coli*, *S. aureus*, *Klebsiella* spp., *Proteus* spp. showed colourless, pink, golden yellow, blue and light brown colour, respectively, on the other hand, changing the medium as blood agar medium they showed colourless, pink to red, yellow, purple and fade brown, respectively. Moreover their colony showed different morphological structures uniquely.

**Table 2.** Frequency and percentage of bacterial isolated from postsurgical wound infection

Name of organism	Frequency	Percentage
*P. aeruginosa*	90	46.15
*E. coli*	53	27.17
*S. aureus*	22	11.28
*Klebsiella* spp.	18	9.23
*Proteus* spp.	12	6.17
Total	195	100

These bacterial pathogens have been found in different frequencies. Among the isolated bacterial pathogens responsible for postsurgical wound infection, 46.15 % were *P. aeruginosa*, 27.17 % were *E. coli*, 11.28 % were *S. aureus*, 9.23 % were *Klebsiella* spp. and 6.17 % were *Proteus* spp. *P. aeruginosa* has shown the highest frequency in both males and females, whereas *Proteus* spp. has demonstrated the lowest frequency. In this study, the percentage of male patients suffering from postsurgical wound infection was higher than female patients. However, the bacterial isolates responsible for postsurgical wound infection do not have any correlation with the gender of the respondents.

**Fig. 1.**
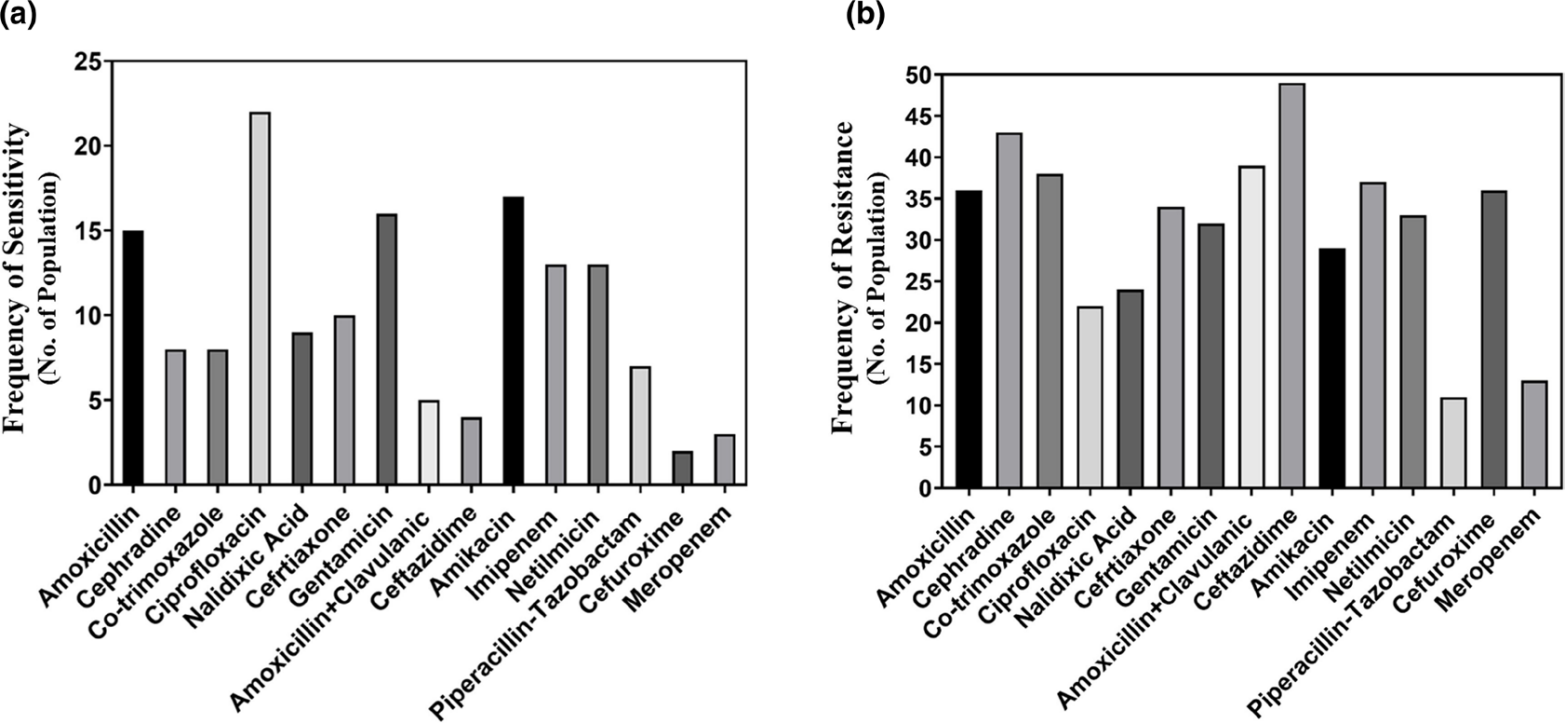
Comparison among the performances of the antibiotics in terms of the frequency of sensitivity (**a**) and frequency of resistance (**b**) against *E. coli.*

A set of antibiotics including amoxicillin, piperacillin-tazobactam, ciprofloxacin, nalidixic acid, gentamicin, cefuroxime, amikacin, netilmicin, cephradine, co-trimoxazole, imipenem, ceftazidime, and amoxicillin+clavulanic acid has been used against *E. coli* in the disc-diffusion method for testing the antibiotic susceptibility. Fig. 1a shows that among the antibiotics used against *E. coli*, ciprofloxacin [9] showed the best performance with the highest frequency of bacterial sensitivity. Amikacin [10] and gentamicin [10] also performed reasonably well. In terms of antibiotic resistance pattern, Fig. 1b reveals that *E. coli* is gaining resistance to several antibiotics like ceftazidime (49), cephradine (43), amoxicillin+clavulanic acid (39) and imipenem (37).

**Fig. 2.**
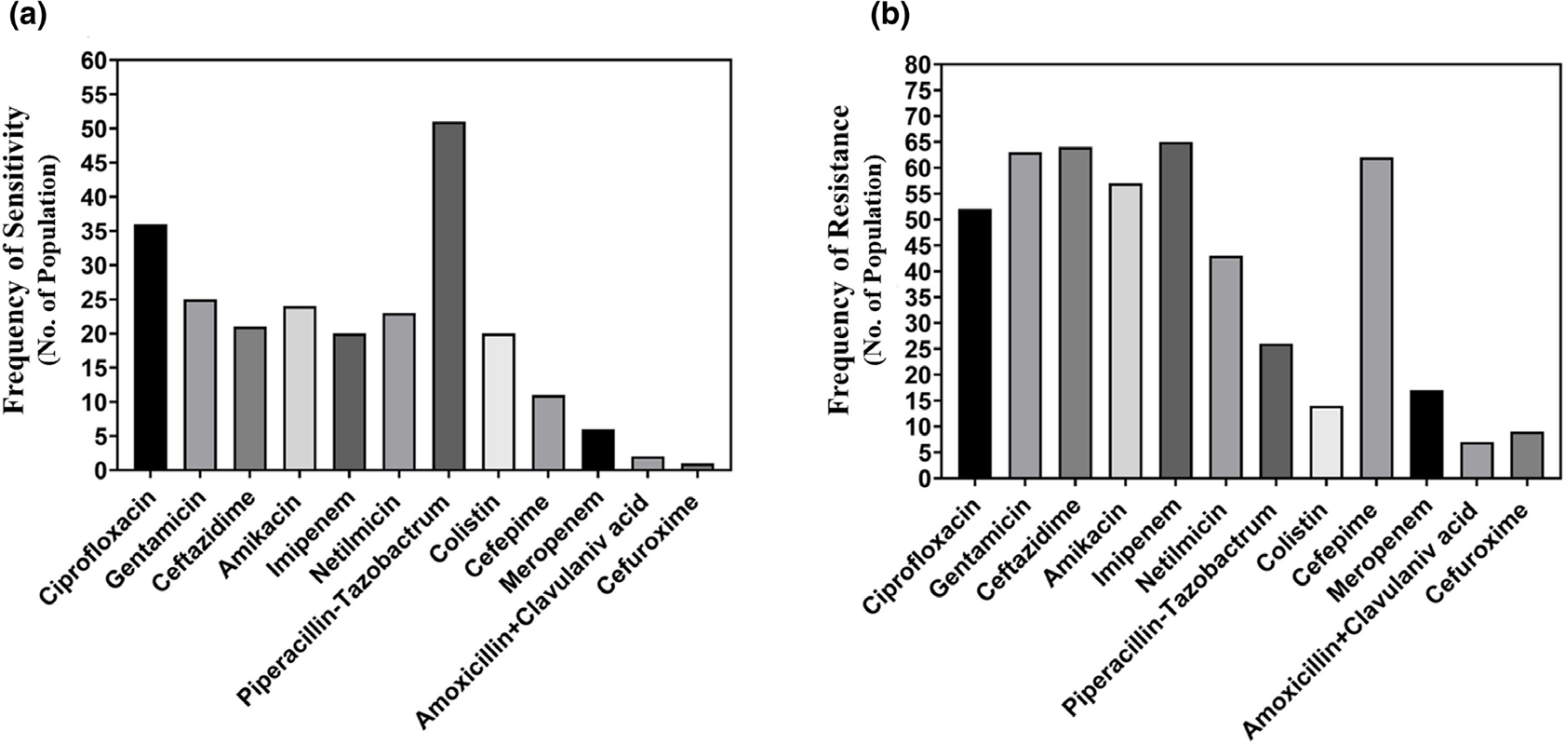
Comparison among the performances of the antibiotics in terms of the frequency of sensitivity (**a**) and frequency of resistance (**b**) against *P. aeruginosa.*

Ciprofloxacin, gentamicin, ceftazidime, imipenem, cefepime, amikacin, piperacillin-tazobactam, colistin and netilmicin have been used against *P. aeruginosa* (Fig. 2). Among the antibiotics used against *P. aeruginosa,* piperacillin-tazobactam showed the best performance with the highest frequency of bacterial sensitivity (51). Ciprofloxacin also performed pretty well (36), and the frequency of bacteria sensitive to this antibiotic was not so high. Fig. 2b shows that the *P. aeruginosa* isolates are resistant to several antibiotics, including imipenem (65), ceftazidime (64), gentamicin (63) and cefepime (62).

**Fig. 3.**
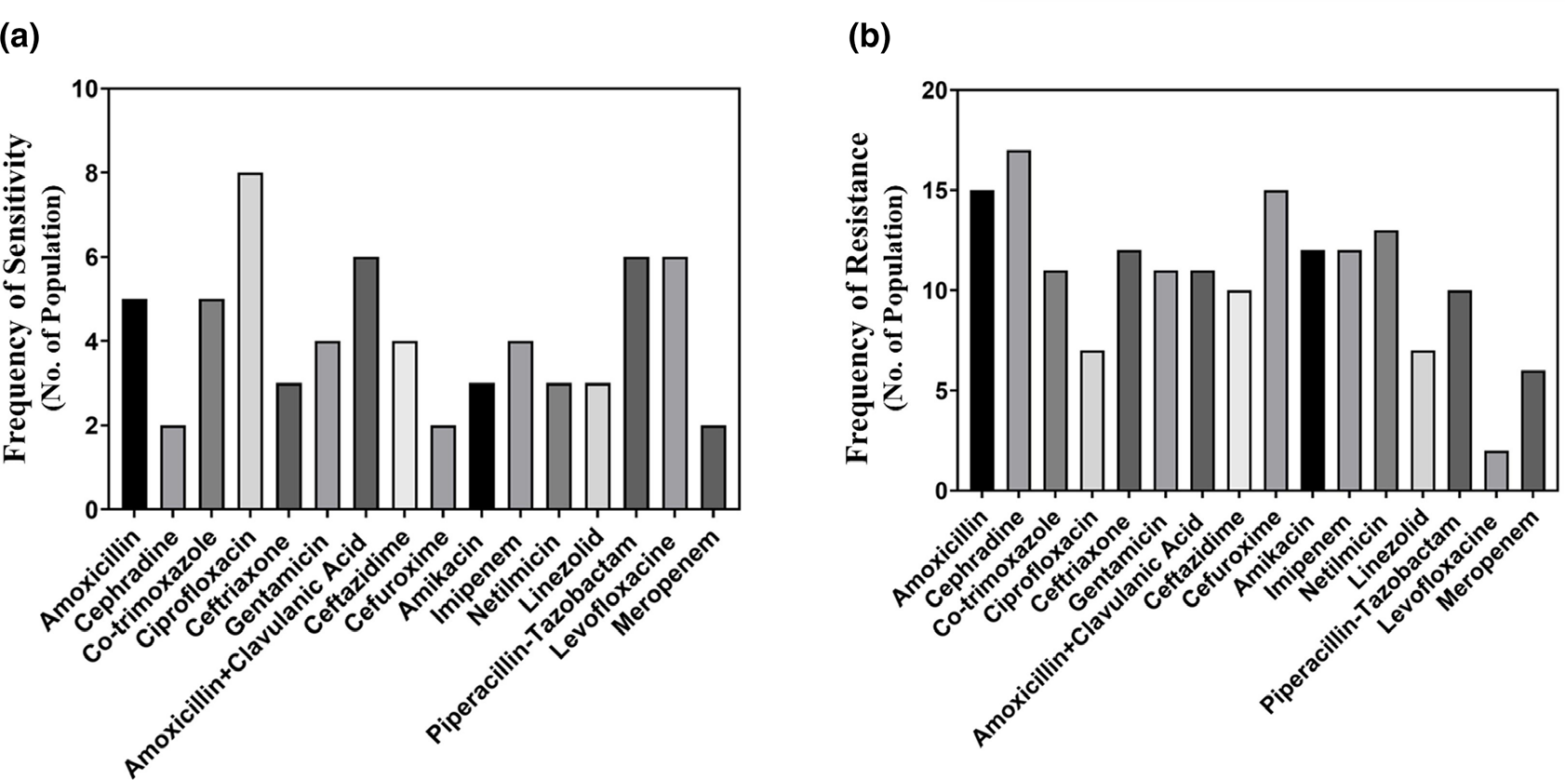
Comparison among the performances of the antibiotics in terms of the frequency of sensitivity (**a**) and resistance (**b**) against *S. aureus*.

Ciprofloxacin [11] and amoxicillin+clavulanic acid [6] performed well against *S. aureus* (Fig. 3a). On the other hand, in Fig. 3b, we can see that *S. aureus* has gained resistance to cephradine [10], cefuroxime [12] and netilmicin [13], which may cause severe complications in the near future.

**Fig. 4.**
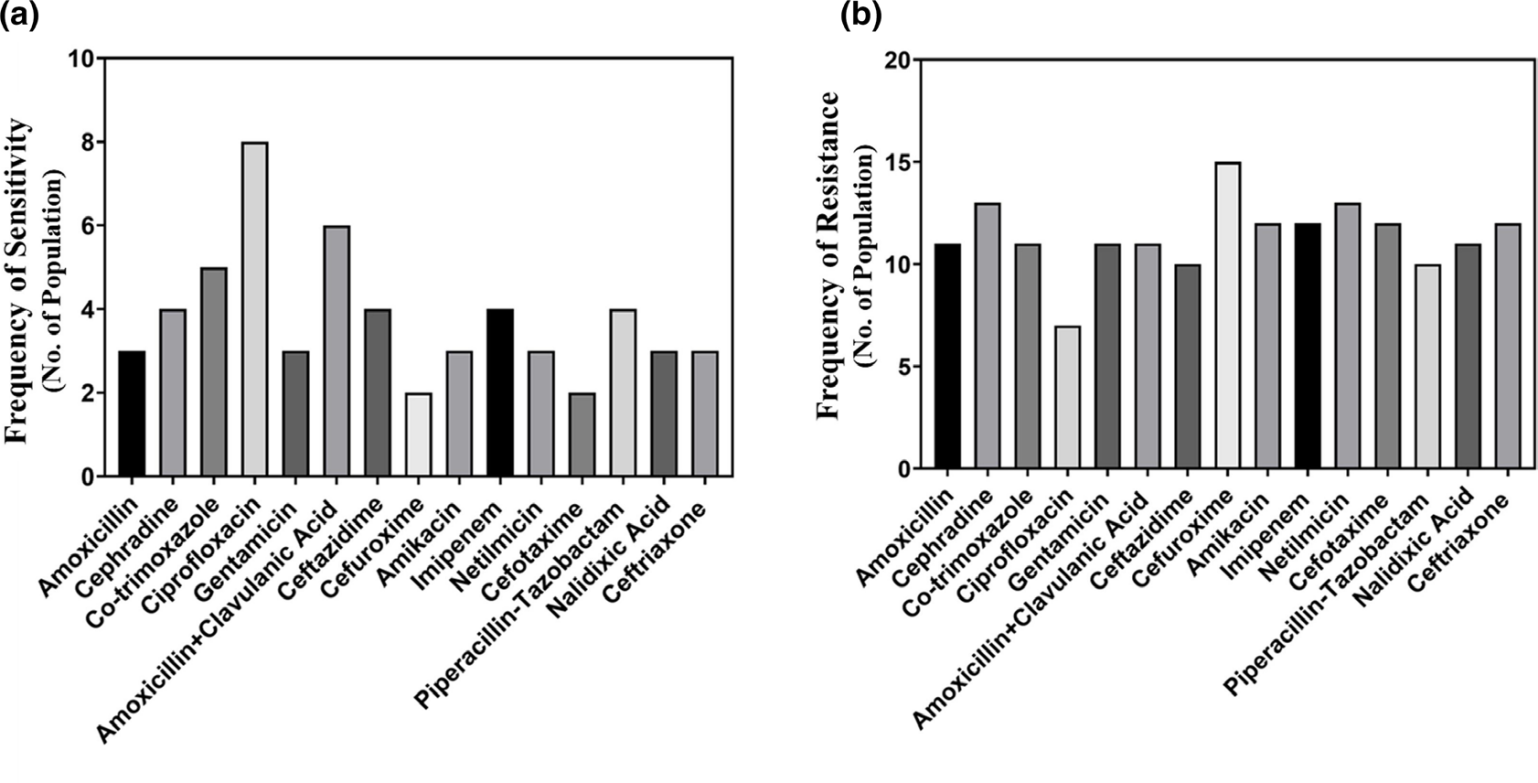
Comparison of the performance of antibiotics in terms of the frequency of the sensitivity (**a**) and resistance (**b**) against *Klebsiella* spp.

The response of *Klebsiella* spp. to different kinds of antibiotics was also evaluated, and the result is depicted in Fig. 4. Ciprofloxacin showed excellent performance against *Klebsiella* spp. with the highest frequency of sensitive bacteria [11]. Amoxicillin+clavulanic acid also exhibited a good performance [6]. However, Fig. 4b reveals the resistance of *klebsiella* isolates to cefuroxime [12], netilmicin [13] and cefotaxime [14], which is fairly concerning.

**Fig. 5.**
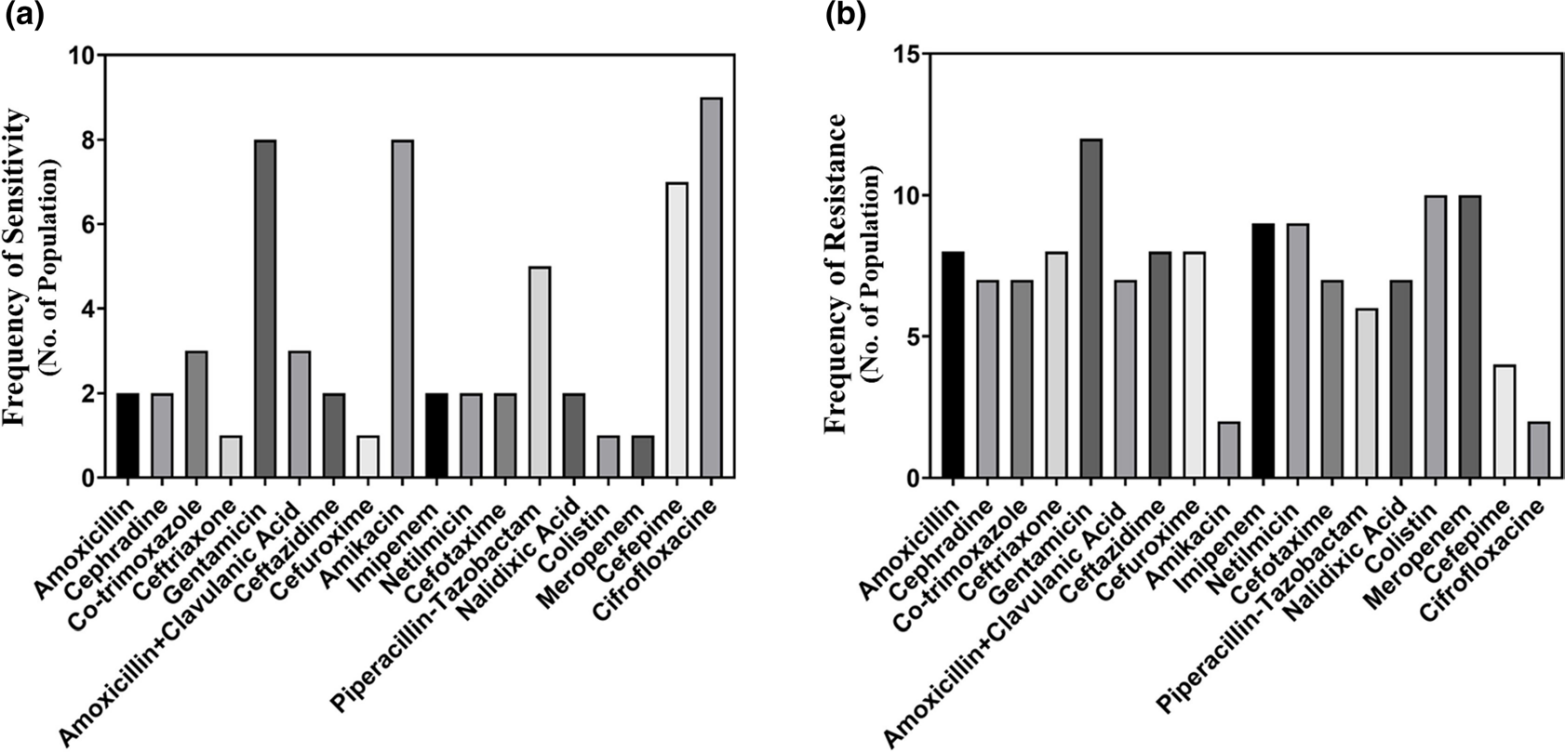
Comparison of the performances of the antibiotics in terms of the frequency of sensitivity (**a**) and resistance (**b**) *Proteus* spp.

All isolates of *Proteus* spp. were found sensitive to ciprofloxacin [15]. Amikacin, gentamicin and cefepime, showed good performance, and they accounted for a reasonable frequency of sensitive bacteria (8, 8 and 7, respectively). Fig. 5b showed resistance of the *Proteus* spp. to colistin and meropenem were ten people. Total nine people of *Proteus* spp. were found resistant to netilmicin.

## Discussion

One of the significant concerns of physicians and surgeons is properly managing and maintaining the patients after surgery to save them from microbial infections. Microbial infections may become worse when antibiotic-resistant micro-organisms are involved. Most government hospitals in Bangladesh do not have enough data regarding the wound infection rate of postsurgical patients. In the current study, bacterial pathogens, including *S. aureus*, *E. coli*, *P. aeruginosa, Klebsiella* species and *Proteus* species were isolated and identified 36, 21.2, 8.8, 7.2 and 4.8 %, respectively, responsible for postsurgical wound infection. Reiye and his co-workers carried out similar work in 2012 in Ayder Teaching and Referral Hospital, Mekelle, Ethiopia, and in that study, those five bacterial pathogens were also identified [11]. Shahin Ara Begum *et al.* studied represented similar bacterial pathogens, but the prevalence of *E. coli, S. aureus, P. aeruginosa, Klebsiella* spp. were 29.41, 27.4, 19.60 and 4.90 %, respectively [3]. The reason beyond the variation of other studies in terms of bacterial pathogens frequencies may be sample size, different regions, different strains of these bacterial species and the time required for the infection. In another study carried out by Agwunglefah and his co-workers in Federal Medical Centre and Christiana Specialist Hospital, Owerri, in 2014, different frequencies of bacterial pathogens were found in male (49 %) and female (23 %) patients suffering from postsurgical wound infections [15]. According to this study, most postsurgical wound infections occurred after hand and abdominal surgeries. In 2015, Sickder and his fellow workers conducted a similar study on the admitted patients and showed laparotomy and hernioplasty had the highest percentage of postsurgical wound infections [16]. Our study observed, that both *E. coli* and *P. aeruginosa* have been found to exhibit higher frequencies in hand and abdominal surgeries. The presence of high frequency of *E. coli* and *P. aeruginosa* indicates the hospital’s unhygienic environments [14, 17]. According to the findings from the studies carried out in Kenya and Ethiopia, *S. aureus* and *Klebsiella* spp. are also prevalent and consistent with our current study’s findings [11].

Several studies have reported that *E. coli* isolates are significantly resistant to certain antibiotics such as tetracycline, chloramphenicol, cephradine, quinolones and others. [12, 13, 18]. But until now, *E. coli* has not shown significant resistance to trimethoprim and ampicillin. In the current study, the *E. coli* isolated were found resistant to ceftazidime, cephradine, amoxicillin+clavulanic acid and imipenem. On the other hand, the result of our study demonstrates that *E. coli* is very susceptible to ciprofloxacin, amikacin and gentamicin. In addition, the result of this study shows that 46.15 % of the bacterial isolates are *P. aeruginosa*, which is consistent with other similar studies [19]. The results of the studies by Oguntibegri and Masaadeh demonstrated that the prevalence of *P. aeruginosa* was 33.3 and 27.78 %, respectively, in their studies [10, 20]. In addition, Stephen *et al.* reported that the prevalence of *P. aeruginosa* isolates was 18.8 % [21]. In this study, the maximum susceptibility of *P. aeruginosa* isolates have been found against piperacillin-tazobactam. In other similar studies, *P. aeruginosa* exhibited higher (88 %) susceptibility to imipenem and meropenem [22]. In the current study, *P. aeruginosa* has shown resistance to antibiotics like imipenem, ceftazidime, gentamicin and cefepime.

Further, the current study found *S. aureus* in the postsurgical wound infection at 11.28 %. Ciprofloxacin showed the best performance against *S. aureus*. and further amoxicillin+clavulanic acid, levofloxacin and linezolid showed good activity. A study conducted at the National Institute of Cardiovascular Diseases (NICVD), Dhaka, showed that *S. aureus* was susceptible to imipenem and cephalexin [23]. Besides, the study conducted in a reputed hospital in Dhaka showed that *S. aureus* was sensitive to linezolid, fusidic acid, vancomycin, amikacin and gentamicin [16]. The resistance data reported from the regional patients of Chittagong, Bangladesh depicted that *S. aureus* was resistant to ampicillin, cephradine and gentamicin [9]. In this work, we have also analysed the data regarding *Klebsiella* spp*.* and evaluated their susceptibility and resistance to antimicrobial agents. Our result reveals that *Klebsiella* isolates *are susceptible to* ciprofloxacin (44.4 %) and amoxicillin+clavulanic acid (33.3 %). In one similar study carried out by Bulbin and his colleagues, *Klebsiella pneumonia* showed 81.6 % susceptibility to ciprofloxacin [24]. Another related research conducted in BIRDEM, Bangladesh on sepsis patients demonstrated that 100 % *Klebsiella* spp. is gentamicin and ampicillin resistant [25].

*Proteus* spp. most often contaminate the wounds and cause postsurgical wound infections. According to our current work ciprofloxacin has shown the best performance with the highest frequency of sensitive bacteria (75 %). A similar result was obtained in another study conducted by Mordi and his co-workers, where *Proteus* spp. were found susceptible to ciprofloxacin and gentamicin [26]. Our study observed that some of the *Proteus* spp. were resistant to colistin, imipenem and netilmicin. Hossain and his fellow workers showed that *Proteus* spp. was resistant to tetracycline, doxycycline and cefuroxime [27]. This study strongly supports the increasing concern about antibiotic resistance in the hospital. It is suggested that the hospital should take proper measures to ensure proper hygiene in the hospital environment. The hospital should also supervise the sensitivity pattern of these pathogens as well as select suitable antibiotic regimens for postsurgical wound infections.

## Conclusion

Our study observed ciprofloxacin as the most potent antimicrobial agent since *E. coli*, *Klebsiella* spp. and *Proteus* spp. were 41.55, 44.4 and 75 %, respectively, sensitive to this antibiotic. Moreover, gentamycin and linezolid have shown better activity on to *S. aureus* (83.3 %) whereas this pathogen is mainly resistant to cefuroxime. At the same time, our result has exhibited that *Klebsiella* spp. is also highly resistant to cefuroxime (83.4 %). The antibiotic susceptibility patterns in this study point to the roles that antibiotic play in the management of postsurgical wound infections in hospitals. Moreover, the resistance data reflect several antimicrobial agents’ limitations in treating such bacterial infections.
